# Reductive Fractionation of Flax Shives in Ethanol Medium over RuNi Bimetallic Catalysts

**DOI:** 10.3390/ijms241411337

**Published:** 2023-07-12

**Authors:** Angelina V. Miroshnikova, Valentin V. Sychev, Valery E. Tarabanko, Aleksandr S. Kazachenko, Andrey M. Skripnikov, Anna O. Eremina, Yuriy Kosivtsov, Oxana P. Taran

**Affiliations:** 1 Institute of Chemistry and Chemical Technology, Krasnoyarsk Science Center, Siberian Branch, Russian Academy of Sciences, Akademgorodok 50, Bld. 24, 660036 Krasnoyarsk, Russia; miroshnikova.av@icct.krasn.ru (A.V.M.); sychev.vv@icct.krasn.ru (V.V.S.); and-skripnikov@yandex.ru (A.M.S.); anna.oleg.er@mail.ru (A.O.E.); taran.op@icct.krasn.ru (O.P.T.); 2 Department of Non-Ferrous Metals and Materials Science, Siberian Federal University, pr. Svobodny 79, 660041 Krasnoyarsk, Russia; 3 Department of Biotechnology, Chemistry and Standardization, Tver State Technical University, 22 nab. A. Nikitina, 170026 Tver, Russia; kosivtsov@science.tver.ru

**Keywords:** flax shives, lignin, bimetallic catalyst, Ru, Ni, CMK-3, fractionation, hydrogenation, propanol guaiacol, methoxyphenols

## Abstract

The reductive catalytic fractionation of flax shives in the presence of bimetallic NiRu catalysts supported on oxidized carbon materials (CM) such as mesoporous Sibunit and carbon mesostructured by KAIST (CMK-3) was studied. The catalysts based on CMK-3 were characterized by a higher surface area (1216 m^2^/g) compared to the ones based on Sibunit (315 m^2^/g). The catalyst supported on CMK-3 (10Ni3RuC400) was characterized by a more uniform distribution of Ni particles, in contrast to the Sibunit-based catalyst (10Ni3RuS450), on the surface of which large agglomerated particles (300–400 nm) were presented. The bimetallic catalysts were found to be more selective towards propanol-substituted methoxyphenols compared to monometallic Ru/C and Ni/C catalysts. A high yield of monomers (up to 26 wt%, including 17% 4-propanol guaiacol) was obtained in the presence of a 10Ni3RuC400 catalyst based on CMK-3.

## 1. Introduction

The extensive use of the fossil resources, such as oil, coal and natural gas, leads to their gradual depletion and environmental problems, mainly associated with greenhouse gases emissions, requiring the development of new methods for the use of renewable plant materials, in particular, using the agricultural waste [1].

Flax shives are the main waste product (up to 70 wt%) in flax cellulose fiber production and represent a lignified part of the stem. Flax shives contain about 25% lignin, 50% cellulose and 20% hemicelluloses, depending on the maturity of the plants, their place of growth and the part of the flax stem [1,2].

The reductive catalytic fractionation (RCF) of lignocellulosic biomass is a strategy to achieve the depolymerization of lignin along with the formation of liquid hydrocarbons while maintaining the carbohydrates complex of hemicelluloses and mainly cellulose for further utilization [3,4,5]. RCF processes use solid bifunctional catalysts containing acid and metal active sites [6,7,8].

RCF processes are carried out under harsh conditions (high temperature, pressure and acidity of the reaction medium) [9]. An important process parameter is the stability of the catalyst, which depends mainly on the surface functionality, the support nature and the metal-support interaction [10]. Materials such as Al_2_O_3_, SiO_2_, TiO_2_ and carbon are widely used as catalyst supports in biomass conversion processes [11,12,13,14]. Carbon as a catalyst support has a number of advantages, such as a high resistance to basic and acidic media and a high stability in hydrothermal conditions, as well as a large surface area and the possibility to improve its chemical surface properties by adding surface functional groups [10].

Previously, in the hydrogenation of flax shives, bifunctional Ni and Ru catalysts on an oxidized carbon support Sibunit showed high activity and provided an increase in the yield of monomeric products from 1.6 to 10 and 12 wt%, respectively [15,16]. Higher yields of monomeric products (~25 wt%) were obtained by Anderson et al. [14] via the RCF of corn stover with Ru/C and Ni/C catalysts. A very efficient RCF of birch wood meal using a Pd/C catalyst produced monophenolics (37 wt% on lignin) and cellulose for subsequent enzymatic conversion that produced 87 wt% glucose yield [5].

Catalyst optimization can be carried out, combining various metals, supports, and promoters. The use of bimetallic catalysts is a promising method for biomass feedstock upgrading, as the interaction between metals can modify the surface properties of the catalyst [17].

The activity of NiRu, NiRh and NiPd catalysts exceeds that of single-component catalysts in the hydrogenolysis of lignin C–O bonds [18]. The NiRu catalyst contains 85% Ni and 15% Ru, composed of Ni surface-enriched and Ru–Ni atomically mixed, ultrasmall nanoparticles showing high activity under low temperature (100 °C) and a low H_2_ pressure (1 bar) in the β-O-4 type C–O bond hydrogenolysis of model compounds. The yield of monomers in the process of hydrogenation of the model compound on a bimetallic catalyst was 58% versus 36 and 8% on monometallic Ni and Ru, respectively [18].

The Pd/Zn/C bimetallic catalyst demonstrated high efficiency in the depolymerization of native poplar wood lignin in a methanol medium [19] and promoted the formation of 4-propyl guaiacol and 4-propyl syringol as the main products, providing a total yield of monomers of 54 wt%. Ni-M bimetallic catalysts (M = Ru, Rh and Pt) showed good activity in the depolymerization of birch wood organosolv lignin [18]; the NiRu catalyst containing 85% Ni and 15% Ru showed the highest activity. The yield of the monomeric product on the Ni85Ru15 catalyst attained 6.8 wt%, and it was seven times higher compared to the result on a monometallic Ru catalyst [18].

The goal of this work is to study the possibilities of the hydrogenation of flax shives on bimetallic NiRu catalysts on various carbon supports.

## 2. Results and Discussion

### 2.1. Characteristics of Catalysts

The analysis of textural characteristics obtained by the method of low-temperature nitrogen adsorption revealed that the oxidative treatment of CM led to a slight increase in the specific surface, and the deposition of metals resulted in the partial blocking of support pores, leading to a moderate decrease in the S_BET_ value (Table 1). The pore diameter of the support also slightly decreased during its oxidation. The acidity of the catalysts was estimated by the point of zero charge [20], and it increased as a result of the support oxidation and decreased after the deposition of metals.

**Figure 1:**
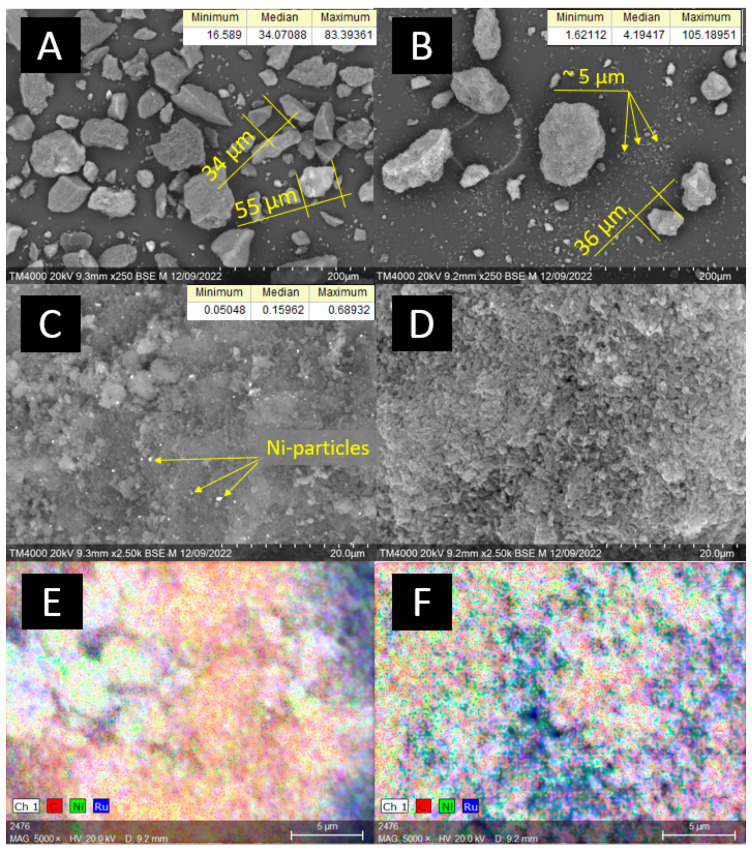
SEM images: (**A**,**C**)—10Ni3RuS450, (**B**,**D**)—10Ni3RuC400; EDX mapping: (**E**)—Ni, Ru on the surface of 10Ni3RuS450, (**F**)—Ni, Ru on the surface of 10Ni3RuC400.

The catalysts’ characterization via scanning electron microscopy (SEM) (Figure 1) revealed that the median size of Sib-4 grains was 34 µm, and for CMK-3 it was 5 µm. CMK-3 was characterized by a more loosened morphology compared to the graphite-like Sibunit. The majority of CMK-3 grains were small particles (<10 µm), but individual agglomerates larger than 100 µm were also observed. The average size of Ni particles in the 10Ni3RuS450 catalyst was 159 nm, and large agglomerated particles (300–400 nm) were also present on the surface. The particle size of Ni on CMK-3 was significantly smaller compared to Sibunit and could not be determined by available SEM equipment. EDX mapping revealed finely dispersed Ni and Ru on both catalysts 10Ni3RuS450 and 10Ni3RuC400.

**Figure 2:**
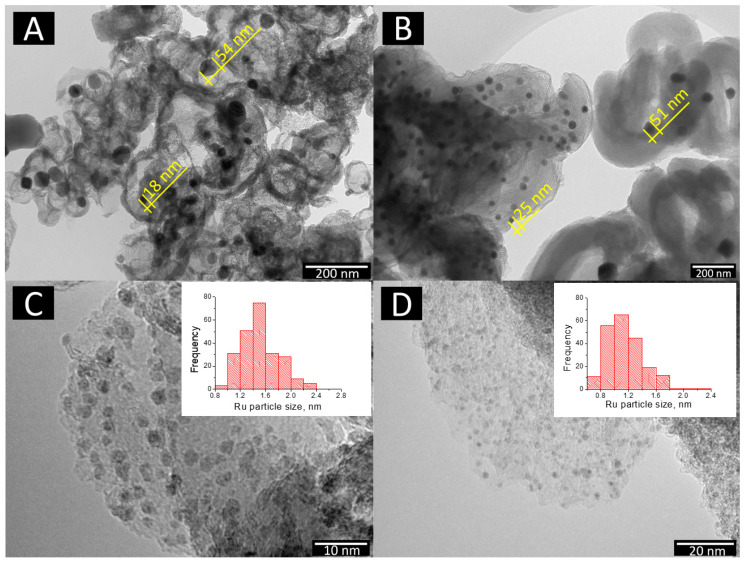
TEM image: nickel particles on the surface—(**A**) 10Ni3RuS450 and (**B**) 10Ni3RuC400; ruthenium particles on the surface—(**C**) 10Ni3RuS450 and (**D**) 10Ni3RuC400.

The transmission electron microscopy study showed that the main volume of nickel on the surface of bimetallic catalysts 10Ni3RuS450 and 10Ni3RuC400 (100–400 nm) was of particles <60 nm in size (Figure 2). Nickel in the 10Ni3RuC400 catalyst was distributed more evenly, perhaps due to the higher acidity of the support or the larger specific surface area of the CM. The same pattern applied to Ru distribution; the particle size of ruthenium on the surface of 10Ni3RuC400 was smaller than that on 10Ni3RuS450, and the dispersion was higher (Table 2, Figure 2).

**Figure 3:**
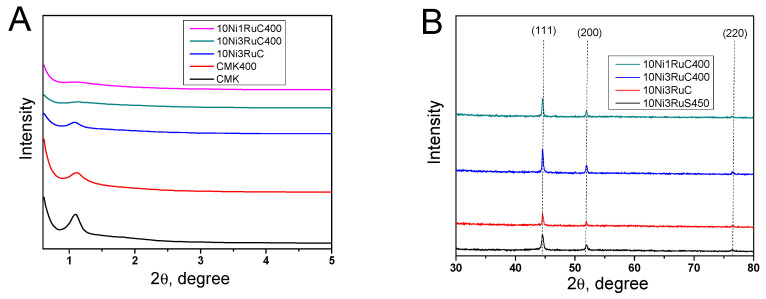
X-ray patterns of carbon supports and bimetallic catalysts: (**A**)—low angle region, (**B**)—high angle region.

The X-ray diffraction patterns of the catalysts showed reflections of (111), (200), and (220) of the metallic form of nickel at 2θ angles of 44, 52, and 76, respectively (Figure 3). The formation of the NiRu alloy phase with (100), (111), and (101) reflexes at 2θ angles of 40, 42, and 46, respectively, was not detected, and hence the alloy was not formed. The low-angle region exhibited a reflex of the mesostructure of CM CMK-3. The ordered structure was loosened a little as a result of the oxidative treatment and the deposition of metals that was revealed by a smoothing of the characteristic peak in the 2θ region of 1.0–1.2. The nickel particle size was calculated using the Scherrer equation on the basis of the (200) reflection. The nickel particle size decreased in the series 10Ni3RuS450–10Ni3RuC–10Ni3RuC400–10Ni1RuC400, which was attributed to the higher acidity of the support and the larger specific surface area of the CM, and which corresponded well with data obtained via SEM and TEM (Table 2).

**Figure 4:**
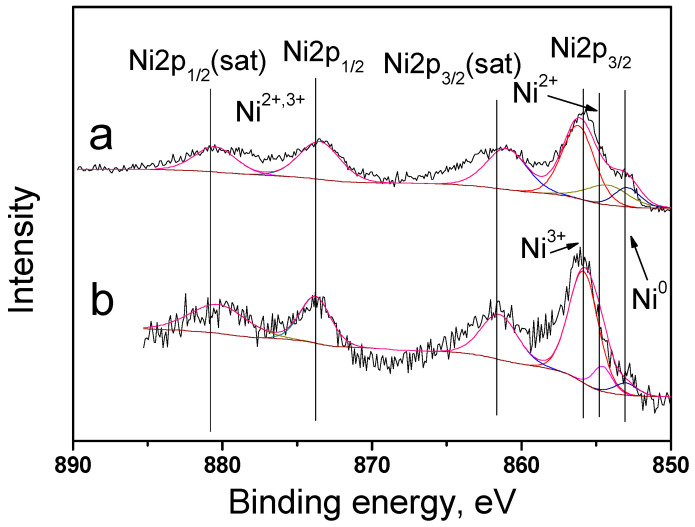
XPS spectra of Ni2p catalysts: (**a**)—10Ni3RuS450, (**b**)—10Ni3RuC400.

The catalyst surface was studied in detail using XPS. Bimetallic catalysts 10Ni3RuS450 and 10Ni3RuC400 were characterized by peaks in the regions of 852.9, 854.5, and 855.8 eV, corresponding to the states of nickel Ni^0^, Ni^2+^, and Ni^3+^, and the ratio of metallic and oxidized forms of nickel is presented in Table 2 [21]. The catalyst surfaces enriched by ruthenium and the surface concentrations of nickel were close to the calculated ones (Figure 4).

**Figure 5:**
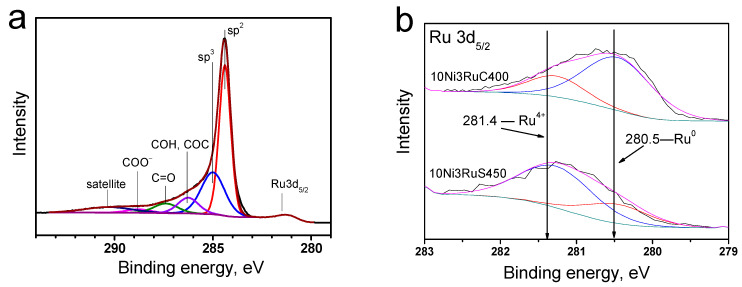
XPS spectra: (**a**)—characteristic C1s for a series of catalysts, (**b**)—Ru3d_5/2_ level for catalysts.

In the region of 284–290 eV, the characteristic peak of graphite was the most intense; the spectrum also contained peaks of amorphous carbon, carbonyl, hydroxyl, and ether groups, as well as fragments of carboxyl groups (Figure 5a) [22].

The analysis of the photoelectron spectra of the Ru3d_5/2_ level (Figure 5b) showed that ruthenium was predominantly oxidized on the surface of the 10Ni3RuS450 catalyst. This is evidenced by the binding energy of the Ru3d_5/2_ peak—281.3 ± 0.1 eV—which is typical for the Ru^4+^ state [23,24]. It should be noted that the range of binding energies for metallic ruthenium is 280.0–280.6 eV. Nevertheless, an analysis of the peak shape of the Ru3d_5/2_ line showed that an asymmetry was observed on the side of lower binding energies, which permitted the conclusion that ruthenium is present in the samples in the metallic state, as well. Using the Casa XPS program, the Ru3d_5/2_ peak was deconvoluted into separate spectral components (Figure 5b), describing metallic and oxidized ruthenium, and their ratio is presented in Table 3. The signal in the region of 280.5 ± 0.1 eV (Figure 5) showed that the main ruthenium state on the surface of the 10Ni3RuC400 catalyst was the metal [23,24].

### 2.2. Analysis of Hydrogenation Products

The yields of liquid products (up to 35%) of flax shives’ hydrogenation on bimetallic catalysts 10Ni3RuS450 and 10Ni3RuC400 were close to the results obtained over the 10NiS450 catalyst (Table 4).

The analysis of the conversion of the flax shives’ main components (Table 5) shows that the yield of cellulose increased under the action of the ruthenium component of the catalyst and decreased when CMK-4 was used instead of Sibunit. A high delignification (90%) was attained on 10NiS450 and 10Ni3RuC400 catalysts, and the second catalyst was the most efficient for obtaining high cellulose yields and delignification degree.

### 2.3. Analysis of Liquid Products

Table 6 shows the yields and composition of the monomer fractions of liquid products. It may be noted that the yield of methoxyphenols on the 10Ni3RuS450 bimetallic catalyst (18 wt%) coincided with the sum of the yields on analogous monometallic catalysts, and more yields of up to 26 wt% were obtained only if using the CMK-3 support.

While going from monometallic Sibunit catalysts to the bimetallic one, the yields of the largest part of the monomeric products decreased, except for 4-propanol guaiacol and syringol, and they became the main products (15% of 18% total yield, Table 6). This may be connected with more monometallic catalysts’ acidity compared to bimetallic ones (pH_PZC_ increased from 7.1 to 8.2). The lower acidity of the 10Ni3RuS450 prevents the acid-catalyzed dehydration of the alcohols, and they become the main products.

**Figure 6:**
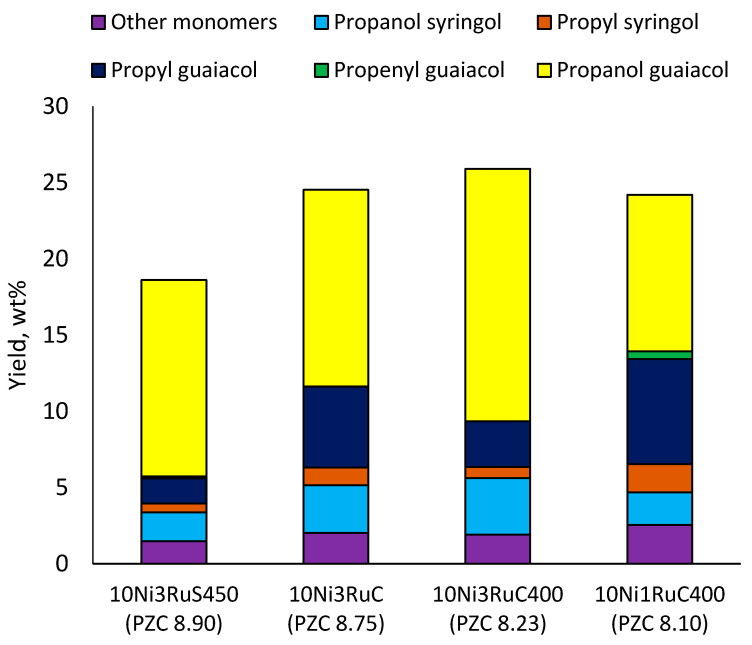
Influence of the catalyst support on the yield of the main monomers.

Among the bimetallic catalysts in the hydrogenation of flax shives, the best results were obtained on the 3Ru10NiC400 catalyst supported on CM CMK-4 oxidized at 400 °C. The yield of monomers on this catalyst attained 26 wt% (Table 5, Figure 6), and it was very close to the known data on the corn stove RCF, 25% [14]. Our catalyst was highly selective for propanols; the content of 4-propanol guaiacol was 17 wt%, and the propanol syringol was 3.7%. Previously, such a 4-propanol guaiacol yield (up to 16 wt%) was attained by hydrogenating the abies wood with a NiCuMo/SiO_2_ catalyst [25]. The higher yield of monophenols (25%) was attained via the fractionation of the wheat straw over the Ru/C catalyst [26] and corn stover fractionation with Ru/C and Ni/C [14]. On the other hand, the Ru/Al_2_O_3_ catalyst gave a lower yield of monomers [26]. The authors ascribed this result to the less favorable interaction of lignin fragments with metal particles supported on Al_2_O_3_ [27] or to the higher tendency of catalysts supported on Al_2_O_3_ to deactivate [26].

The increase in the activity of the catalysts based on CMK-3 was probably caused by the significantly higher specific surface area and smaller grain size of the catalysts. This led to an increase in the rate of inner diffusion, due to which the rate of condensation of the products and intermediates of the process decreased, i.e., the yield of monophenols increased. An increase in the yields of monomeric products and, first of all, of 4-propanol guaiacol with an increasing efficiency of mass transfer due to an increase in the stirring rate and a decrease in the grain size of the catalyst was shown earlier [15]. The high activity of more dispersed catalysts on CMK-3 and their selectivity for guaiacyl and syringyl propanols corresponded to this dependence. The high activity of the catalyst supported on CMK-3 compared to the catalyst on Sibunit could also be associated with the domination of ruthenium metallic form in the first case (Figure 5b).

To conclude, let us compare the maximum yields of methoxyphenols in the hydrogenolysis process (25 wt%) with the known data [28] on the maximum total yields of vanillin and syringaldehyde during the oxidation of flax shives with nitrobenzene (19–21%). Note that the yields of aromatic aldehydes in the processes of lignin oxidation with nitrobenzene are considered to be the theoretical limit for the yield of monophenols in the oxidation processes caused by the structure of the oxidizing lignin [29]. Taking into account the difference in the molecular weights of vanillin and guaiacyl propanol, the main products of hydrogenation and oxidation, the molar ratio of the yields of these products was reduced to a value of 1.04, very close to unity. The closeness of the yields of methoxyphenols in these fundamentally different processes of chemical degradation of the polymeric structure of lignins shows that in the studied process of the hydrogenation of flax shives on the bifunctional bimetallic catalyst 10Ni3RuC400, the maximum possible selectivity for the sum of methoxyphenols was attained.

### 2.4. The Influence of the Supports’ Acidity on the Yield of Monomeric Products

The results obtained (Figure 6) demonstrate the extreme dependence of the yield of monomeric products on the acidity of the catalyst in the range of pH_PZC_ 8.1–8.9 with a maximum at pH_PZC_ 8.2.

**Figure 7:**
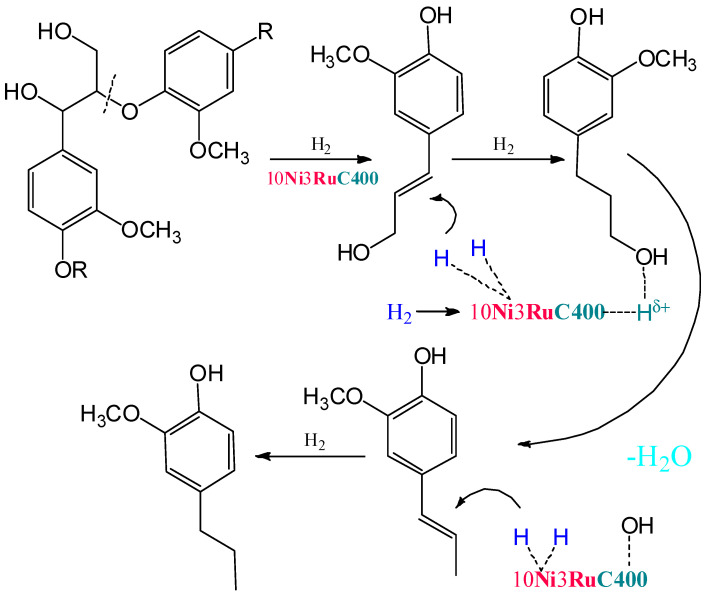
The scheme of lignin hydrogenation in the presence of bifunctional bimetallic catalysts.

Within the previously postulated scheme of lignin hydrogenation [16] (Figure 7), the metal sites of the catalyst provide the hydrogenation of double bonds, while the acid sites provide the dehydration of alcohol structures to olefins. Acid centers can also catalyze condensation processes, which leads to a decrease in the yields of monomeric products. The competition of these two processes leads to an extreme dependence of the yield of products on the acidity of the catalysts. A similar extreme dependence was shown for ruthenium monometallic catalysts [30].

### 2.5. The Catalyst Recycling

**Figure 8:**
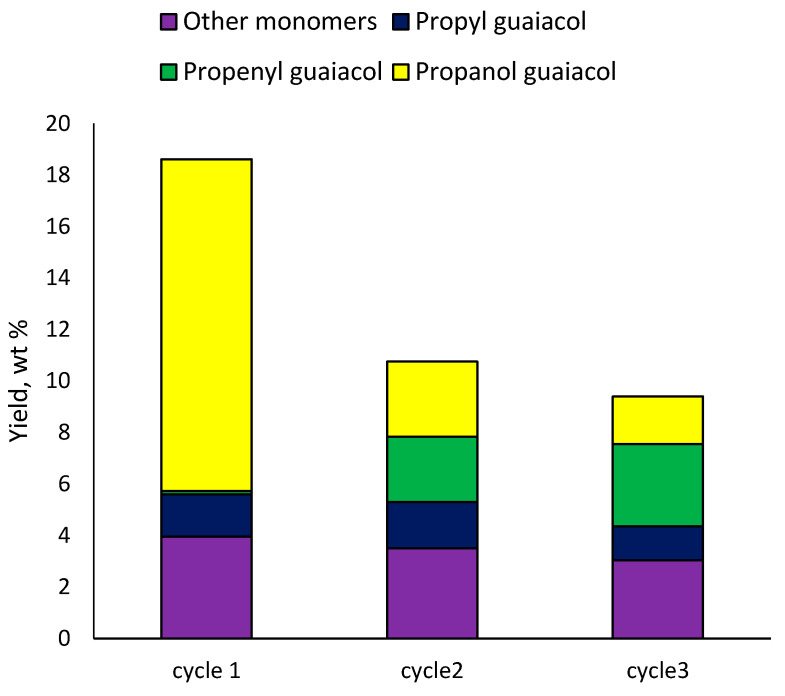
Effect of 10Ni3RuS450 catalyst recycling on the yield of the main methoxyphenols.

The 3Ru10NiS450 catalyst was separated from the solid residue using a magnet, followed by sifting through a sieve (cell 100 μm). The results of the catalyst recycling revealed that after the first cycle the monomer yield decreased from 18 to 11 wt% (Figure 8). In the second cycle, the decrease was less significant (down to 9 wt%). First of all, when the catalysts were recycled, their hydrogenating activity decreased, since the yield of 4-propenyl guaiacol increased from 0.1 to 2.5–3 wt%. At the same time, the content of 4-propanol guaiacol was significantly reduced; its yield did not exceed 3 wt%. Such results can be explained by the fact that the acidity of the catalyst was maintained, and the hydrogenation activity decreased. Data from Table 5 show that the ruthenium catalyst had greater hydrogenating and dehydrating activity compared to the nickel catalyst. Therefore, the activity of the ruthenium component was probably lost in the second and third cycle.

An analysis of the solid residue of hydrogenation showed that after the first catalyst recycling, the cellulose yield decreased by a factor of 1.5 (to 38.5 wt%), and with the subsequent recycling the yield of components and the degree of delignification barely changed (Table 7).

## 3. Materials and Methods

### 3.1. Flax Shives Samples Preparation

The flax shives (growing region—Tver region, Russia) contained (% of the absolutely dry substrate weight) cellulose (50.6), lignin (30.4), hemicelluloses (17.1), and ash (1.9). Flax shives had a fraction size 0.5–2 mm. Moisture content was lower than 1 wt%.

### 3.2. Preparation of Catalysts

Oxidized carbon samples were prepared from commercial graphite-like mesoporous carbon material (CM) Sibunit-4 (S4) and mesostructured CM CMK-3 (carbon mesostructured by KAIST), oxidizing them with oxygen in a mixture of 20 vol.% in N_2_ in the presence of water vapor (70.1 kPa) at a given temperature (400, 450 °C) for 2 h [31]. The catalysts were prepared using the layer-by-layer deposition method. Nickel was deposited with incipient wetness impregnation (IWI) using an aqueous solution of nickel (II) chloride hexahydrate (NiCl_2 6_H_2_O) followed by drying at room temperature for 3 h and at 60 °C for 12 h. The reduction of the active component was carried out in a quartz reactor in a hydrogen flow (flow 30 mL/min) at 450 °C for 2 h, the temperature was increased at a rate of 8 °C/min, after cooling to room temperature in a hydrogen atmosphere, and the catalyst was passivated with a gas mixture containing 1% of O_2_ in N_2_ (flow 200 mL/min, 0.5 h) [32,33]. Ru deposition was also carried out with the IWI technique using an aqueous solution of Ru(NO)(NO_3_)_3_ followed by sample drying at room temperature for 2–3 h and at 60 °C for 12 h. The active component was reduced in a hydrogen stream (30 mL/min) at 300 °C for 2 h (temperature ramp: 1 °C/min). After cooling down to room temperature under hydrogen, the catalyst was passivated using a gas mixture of 1% of O_2_ in N_2_ flow 30 mL/min [32].

The surface morphology was studied using a Hitachi TM4000 Plus scanning electron microscope with an attachment for energy-dispersive microanalysis for a qualitative and quantitative X-ray spectral microanalysis of the samples’ composition.

High-resolution electron micrographs of the catalysts were obtained with a Hitachi HT7700 transmission electron microscope (Hitachi, Tokyo, Japan, 2014) at an accelerating voltage of 110 kV and a resolution of 2 Å. Particle size distribution histograms were obtained as a result of statistical (500–800 particles) processing. The mean linear particles size (<d_l_>) and mean surface volume particle size (<d_s_>) were calculated using the Formulas (1) and (2):

<d_l_> = Σd_i_/N, (1)

<d_s_> = Σd_i_^3^/Σd_i_^2^, (2)

where d_i_—measured diameter of a Ru particle; N—the total number of Ru particles.

Dispersion of Ru (D_Ru_) in prepared catalyst was calculated using Formula (3):

(3) $$D_{\mathrm{Ru}}=6\cdot \frac{M_{\mathrm{Ru}}}{\alpha_{\mathrm{Ru}}\cdot \rho \cdot N_{0}\cdot u\text{<}d_{s}\text{>}},$$

M_Ru_ is the atomic weight of ruthenium (0.101 kg/mol), ρ is the density of ruthenium (12,410 kg/m^3^), α_Ru_ is the average effective area occupied by a Ru atom on the surface (6.13 × 10^−20^ m^2^), N_0_ is the Avogadro number, and d_s_ is the mean volume-surface particle size [30].

Photoelectron spectra were recorded on a SPECS spectrometer with a PHOIBOS MCD9 hemispherical energy analyzer under excitation by monochromatic Al Kα radiation, electron collection angle 90°. The element concentrations were determined from the survey spectra. When decomposed using the CasaXPS package, the non-linear Shirley background was subtracted and the Gaussian–Lorentzian shape of the peaks was used.

Powder diffraction data were obtained using CuKα radiation on an X’Pert PRO diffractometer with a PIXcel (PANalytical) detector equipped with a graphite monochromator. The sample was ground in an agate mortar and prepared by powdering. The surveys were carried out at room temperature in the small-angle range from 5 to 80 °C on the 2θ scale, in steps of 0.026 °C, ∆t—50 s.

Quantitative elemental analysis of the obtained catalysts was carried out on an AxiosAdvanced X-ray fluorescence spectrometer (PANalytical, Almelo, The Netherlands). For analysis, the test material was pressed together with boric acid H_3_BO_3_ as a binder into a tablet with a diameter of 32 mm.

The acidity of the catalysts was evaluated by the point of zero charge (PZC) according to the Sorenson–de Bruyn method [20]. A total of 10 mL of distilled water was placed in a potentiometric cell, then successively in small portions (0.01 g); the conditions were not accurately characterized, and the stoichiometry was added to the test sample with continuous stirring with a magnetic stirrer at time intervals of 5–10 min until a constant potential of the glass electrode was reached [20].

### 3.3. Hydrogenation of Flax Shives

Flax shives were hydrogenated using a ChemRe SYStem R-201 autoclave (ChemRe SYStem R-201, Anyang, Republic of Korea, 2017) (300 mL vessel). The reactor was loaded with 60 mL (1.05 mol) of ethanol, 3.0 g of the substrate, and 0.3 g of the catalyst, similar to the procedure in [16]. Initial hydrogen pressure was set to 4 MPa. The reaction was carried out at 225 °C for 3 h with 1000 rpm stirring. In the reaction course, the pressure in the reactor ranged from 9.1 to 11.5 MPa, depending on the process conditions. After completing the process, the products were separated and analyzed according to [16].

The liquid product yield (wt%) was calculated as:

$$a_{1}=\frac{m_{l}}{m_{\mathrm{fs}}}\times 100\%,$$

where m_1_ is the liquid product weight (g) and m_fs_ is the weight of flax shives (g).

The solid residue yield was calculated as:

$$a_{2}=\frac{m_{\mathrm{sr}}-m_{\mathrm{cat}}}{m_{\mathrm{fs}}}\times 100\%$$

where m_sr_ is the solid residue weight (g) after the extraction and m_cat_ is the catalyst weight (g).

The flax shives’ conversion was determined using the following formula:

$$X_{\mathrm{fs}}=\frac{m_{\mathrm{fs}}-m_{\mathrm{sr}}-m_{\mathrm{cat}}}{m_{\mathrm{fs}}}\times 100\%.$$

The degree of delignification was calculated as:

$$X_{l}=\frac{m_{\mathrm{fs}}-m_{\mathrm{lsr}}}{m_{\mathrm{lfs}}}\times 100\%,$$

where m_lfs_ and m_lsr_ are the weight of lignin in flax shives and in the solid residue (g), respectively.

The cellulose yield (wt%) was determined as:

$$X_{c}=\frac{m_{\mathrm{csr}}}{m_{\mathrm{cfs}}}\times 100\%,$$

where m_cfs_ and m_csr_ are the cellulose weights in flax shives and in the solid residue, respectively.

The yield of monomeric compounds based on lignin was calculated by:

$$Y(\%)=\frac{S_{i}\times C_{\mathrm{st}}}{S_{\mathrm{st}}}\times k/C_{\mathrm{lig}}\times 100$$

where S_i_ is substance peak area, S_st_ is internal standard peak area, C_st_ is internal standard concentration, k is the calibration factor, and C_lig_ is lignin concentration.

The liquid ethanol-soluble products of hydrogenation of the flax shives were analyzed via GC–mass spectrometry (MS) using an Agilent 7890A chromatograph (Agilent, Santa Clara, CA, USA) with an HP-5MS capillary column (30 m) at temperature programming in the range of 40–250 °C and an Agilent 7000A Triple Quad (Agilent, Santa Clara, CA, USA) selective mass spectrometer. The compounds were identified using the NIST MS Search 2.0 instrument database.

## 4. Conclusions

The process of hydrogenation of flax shives on monometallic catalysts supported on Sibunit produced propyl guaiacol as the main product. When switching to a bimetallic catalyst on Sibunit, propanol guaiacol became the main product. This is probably due to the decrease in the acidity of the catalysts upon transition from monometallic to bimetallic (pH_PZC_ increased from 7.1 to 8.2).

When the Sibunit support of the bimetallic catalysts was replaced by CMK-3, the composition of the products did not change significantly, but their total yield increased by almost 1.5 times. This may be due to the larger specific surface area of CMK-3 and the significantly smaller average grain size of the catalysts (5 μm) compared to the catalysts based on Sibunit (34 μm). Another possible reason for increasing the yield was a significant increase in the metallic ruthenium content while transferring from Sibunit to CMK-3. These effects led to the increase in the activity of catalysts based on CMK-3, and to decreasing the diffusion limitation of the processes. They also both reduced the rate of condensation of the products and intermediates of the processes, i.e., increasing the yield of methoxyphenols.

The attempts to recycle the 10Ni3RuS450 catalyst led to a decrease in its activity, mainly the ruthenium component.

The most significant result was obtained by replacing the support of the bimetallic catalyst, Sibunit, with CMK-3, providing uniquely high yields of methoxylated phenolic products of flax shives hydrogenation—25 wt% for lignin. The comparison of this result with the known data on the maximum total yields of vanillin and syringaldehyde during the oxidation of flax shives with nitrobenzene (20%) shows that the molar ratio of the yields of methoxylated products in the hydrogenation and oxidation processes was very close (Y_Hydrogenation_/Y_Oxidation_ = 1.04). The coincidence of the yields of methoxyphenols in these fundamentally different processes of the chemical degradation of the polymeric structure of lignins showed that, in the studied process of the hydrogenation of flax shives on the bifunctional bimetallic catalyst 10Ni3RuC400, the maximum possible selectivity of the process with respect to the sum of methoxyphenols was attained.

## Figures and Tables

**Table 1:** Characteristics of the carbon supports and the catalysts.

No.	Support ^1^/Catalyst	Code	pH_pzc_ ^2^	Textural Properties ^3^			Metal Content ^4^	
				S_BET_, m^2^/g	V_pore_, cm^3^/г	<d_pore_>, nm	Ru	Ni
1	Sib-4	Sib	7.54	375	0.55	5.87	-	-
2	Sib-4-ox450 ^3^	S450	5.33	380	0.53	5.66	-	-
3	CMK-3	CMK	8.54	1390	1.30	3.74	-	-
4	CMK-3-ox-400	CMK400	4.82	1408	1.30	3.70	-	-
5	10% Ni + 3% Ru/Sib-4-ox-450 ^3^	10Ni3RuS450	8.90	315	0.51	5.61	3.0	9.7
6	10% Ni + 3% Ru/CMK	10Ni3RuC	8.75	1315	1.22	3.71	3.1	10.1
7	10% Ni + 3% Ru/CMK-3-ox-400	10Ni3RuC400	8.23	1216	1.00	3.21	3.3	10.4
8	10% Ni + 1% Ru/CMK-3-ox-400	10Ni1RuC400	8.10	1240	1.11	3.22	1.2	9.6
9	10% Ni/Sib-4-ox-450 [15]	10NiS450	8.70	315	0.51	5.61	-	10.0
10	3% Ru/Sib-4-ox-450 [16]	3RuS450	6.89	341	0.50	5.88	2.9	9.8

^1^—Median support granule size: Sib—4–34 µm, CMK—3–5 µm. ^2^—pH_pzc_—pH of the point of the zero charge. ^3^—Textural characteristics were obtained by processing the data of low-temperature nitrogen adsorption. S_BET_—specific surface according to the BET model (m^2^/g), V_pore_—total pore volume (cm^3^/g), <d_pore_>—average pore size (nm). ^4^—metal content obtained via XRF.

**Table 2:** Metal particles’ distribution on various supports.

No.	Code	Ni Particle Size, nm ^1^	Ru Particle Size, nm ^2^				D_Ru_ ^3^
			d_min_	<d_l_>	d_max_	<d_S_>	
1	10Ni3RuS450	32.09	0.77	3.47	1.41 ± 0.02	1.72	0.78
2	10Ni3RuC	30.70	-	-	-	-	-
3	10Ni3RuC400	22.78	0.61	2.81	1.23 ± 0.01	1.49	0.89
4	10Ni1RuC400	21.27	-	-	-	-	-

^1^ The particle size was determined using the Scherrer equation on the basis of the Ni (200) reflection. ^2^ Ru particles sizes obtained via TEM statistical analysis. d_min_, d_max_—minimum and maximum diameter of the particles; <d_l_> = Σd_i_/N—the mean linear particle size; <d_S_> = Σd_i_^3^/Σd_i_^2^—the mean volume_surface particle size. ^3^ D_Ru_—dispersion of Ru.

**Table 3:** The elemental composition of the catalyst surface determined with XPS.

Catalyst	C		O		Ru		Ni		Ni^0^/Ni_ox_	Ru^0^/Ru_ox_
	at%	wt%	at%	wt%	at%	wt%	at%	wt%		
10Ni3RuS450	87.28	71.73	9.16	10.03	1.36	9.43	2.19	8.80	0.15	0.32
10Ni3RuC400	85.11	68.35	10.70	11.44	1.33	8.97	2.86	11.24	0.08	2.50

**Table 4:** Hydrogenation of flax shives in the presence of mono and bimetallic catalysts on various supports.

Catalyst	Products Yield wt%			
	Liquid	Solid	Main Methoxyphenols	Total Monomer Yield
Without Catalyst	36.4	45.4	1.1	1.7
10NiS450 [15]	32.0	34.9	7.8	9.7
3RuS450 [16]	42.5	33.0	10.2	12.2
10Ni3RuS450	35.3	42.6	18.0	18.6
10Ni3RuC *	39.1	47.6	23.6	24.5
10Ni3RuC400	35.2	48.2	25.2	25.9
10Ni1RuC400	42.4	43.8	22.9	24.2

* CMK-3—not oxidized.

**Table 5:** Composition of solid products of flax shives’ fractionation (225 °C, 3 h).

Conditions	Composition of the Solid Residue, wt%			Delignification	Cellulose Yield, wt%
	Hemicelluloses	Lignin	Cellulose		
Without Catalyst	4.2	27.3	68.5	63.3	55.4
10NiS450 [15]	2.1	11.5	42.2	90.6	42.2
3RuS450 [16]	5.8	15.5	79.5	83.2	51.4
10Ni3RuS450	18.4	16.5	65.1	76.9	54.9
10Ni3RuC *	23.1	14.6	62.3	82.1	46.2
10Ni3RuC400	23.6	8.9	67.5	89.3	48.9
10Ni1RuC400	25.2	13.4	61.4	85.3	40.4

* CMK-3—not oxidized.

**Table 6:** The yields of main monomers (225 °C, 3 h).

Substance	Structure	Non	3Ru S450 [16]	10Ni S450 [15]	10Ni3 Ru S450	10Ni3 RuC	10Ni3 RuC400	10Ni1 RuC400
Guaiacol	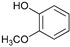	0.36	0.55	0.44	0.28	0.16	0.14	0.21
Methyl guaiacol	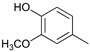	0.01	0.01	0.09	0.12	0.21	0.36	0.32
Ethyl guaiacol	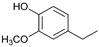	0.21	0.88	0.33	0.33	0.53	0.49	0.53
Propyl guaiacol	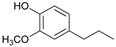	0.08	4.95	2.73	1.65	5.31	2.99	6.91
Propenyl guaiacol	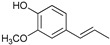	0.19	1.17	1.53	0.13	0.01	0.01	0.49
Ethyl syringol	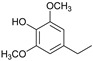	0.04	0.24	0.12	0.11	0.2	0.18	0.19
Propyl syringol	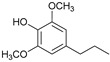	0.03	1.26	0.88	0.58	1.15	0.72	1.85
Propanol guaiacol	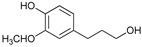	0.08	1.09	1.58	12.88	12.9	16.56	10.27
Propanol syringol	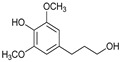	Abs.	Abs.	Abs.	1.88	3.13	3.72	2.14
Propenyl syringol	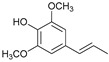	0.14	0.06	0.12	trace	trace	trace	trace
Main methoxyphenols, wt%		1.14	10.21	7.82	17.96	23.6	25.17	22.91
Other monomens		0.52	2.00	1.85	0.65	0.93	0.74	1.29
Total monomer yield		1.66	12.21	9.67	18.61	24.53	25.91	24.2

**Table 7:** The influence of the catalyst recycling on the composition of the solid product of flax shives fractionation (225 °C, 3 h).

Conditions	Composition of the Solid Residue, wt%			Delignification	Cellulose Yield, wt%
	Hemicelluloses	Lignin	Cellulose		
10Ni3RuS450 (1st cycle)	18.4	16.5	65.1	76.9	54.9
10Ni3RuS450 (2nd cycle)	22.4	17.6	60.0	81.2	38.5
10Ni3RuS450 (3rd cycle)	22.9	17.9	52.9	78.4	38.4

## Data Availability

Data are available on request from the authors.
